# Feasibility of Robot - assisted Segmental Ureterectomy and Ureteroureterostomy in Patient with High Medical Comorbidity

**DOI:** 10.1590/S1677-5538.IBJU.2016.0026

**Published:** 2017

**Authors:** Ali Abdel Raheem, Atalla Alatawi, Dae Keun Kim, Abulhasan Sheikh, Koon Ho Rha

**Affiliations:** 1 Department of Urology, Tanta University Medical School, Egypt;; 2 Department of Urology and Urological Science Institute, Yonsei University College of Medicine, Seoul, South Korea;; 3 Department of Urology, CHA Seoul Station Medical Center, CHA University Medical School, Seoul, Republic of Korea

## Abstract

**Introduction and objectives:**

Nephroureterectomy remains the gold standard treatment option for upper tract tumors. However, segmental ureterectomy may be another option in patients with single kidney, borderline renal function or high medical comorbidities. The aim of this video is to assess the feasibility of robotic surgery as a minimally invasive technique in treatment of a high comorbid patient with ureteric tumor.

**Materials and Methods:**

Eighty-year old male patient, with a medical history of chronic hypertensive and uncontrolled Diabetes Mellitus, was referred to our department for treatment of ureteric tumor. Patient underwent robot-assisted radical prostatectomy 5 years ago. Patient’s Charlson comorbidity index score was 9. Computed tomography showed a 2.5cm right ureteral luminal filling enhancing lesion at lower part of upper 1/3 ureter. We performed diagnostic flexible cystoscopy under local anesthesia to exclude associated lower urinary tract carcinoma, and bladder wash was negative for malignancy. Under general anesthesia patient underwent diagnostic flexible ureteroscopy to confirm mass location, and a retrograde pyelography to rule out additional tumors on the right collecting system. Then, the patient was placed in the full lateral flank position without Table flexion. Ports placement were inserted as follow: a “12mm” optical trocar at pararectal line superior and lateral to umbilicus, two “8mm” robotic trocars cranial and caudal to optical trocar (8cm distance), a “8mm” robotic trocar towards anterior superior ischial spine, and a “12mm” assistant trocar was inserted between umbilicus and pubic bone. The surgical steps are shown in the video.

**Results:**

The procedure was performed easily. The total operative time and consol time were 100 and 60 minutes, respectively. Blood loss was 50ml. No reported intraoperative or postoperative complications. Notably, we took full precautions in case of intraoperative failure to complete the procedure successfully, nephroureterectomy was our second option. Postoperative serum creatinine was 1.2mg/dL and length of hospital stay was 2 days. The frozen biopsy showed that the tumor was resected with safe proximal and distal surgical margins. Final histopathology revealed high grade (G3) urothelial carcinoma (pT3), measures (1.3x1.2x0.2cm), associated with carcinoma in situ.

**Conclusion:**

We affirm that robotic segmental ureterectomy and ureteroureterostomy could be offered safely as a minimally invasive treatment for patients with ureteric tumors and high-risk medical comorbidities. It provides excellent perioperative outcomes and early oncological safety with regard to surgical margins.

## ARTICLE INFO

**Available at: http://www.intbrazjurol.com.br/video-section/20160026_raheem_et_al/**

**Int Braz J urol. 2017; 43 (video #10): 779-80**

